# Interdisziplinäres COVID-Board bei SARS-CoV-2-getriggerter hyperferritinämischer Inflammation

**DOI:** 10.1007/s00063-020-00750-8

**Published:** 2020-10-28

**Authors:** P. La Rosée, H.-C. Bremer, F. La Rosée, P. Mohm, A. Hochhaus, I. Gehrke, B. Kumle, A. Benzing, S. Russo

**Affiliations:** 1 Klinik für Innere Medizin II, Hämatologie, Onkologie, Immunologie, Infektiologie und Palliativmedizin, Schwarzwald-Baar-Klinikum, Klinikstr. 11, 78052 Villingen-Schwenningen, Deutschland; 2 Medizinische Fakultät, Universitätsklinikum Jena, Friedrich-Schiller-Universität Jena, Jena, Deutschland; 3 Lungenzentrum, Schwarzwald-Baar-Klinikum, Donaueschingen, Deutschland; 4 Medizinische Fakultät Carl Gustav Carus, Technische Universität Dresden, Dresden, Deutschland; 5 Klinik für Innere Medizin II, Universitätsklinikum Jena, Jena, Deutschland; 6 Klinik für Innere Medizin IV, Innere Medizin Altersmedizin, Schwarzwald-Baar-Klinikum, Donaueschingen, Deutschland; 7 Klinik für Akut- und Notfallmedizin, Schwarzwald-Baar-Klinikum, Villingen-Schwenningen, Deutschland; 8 Klinik für Anästhesiologie, Intensiv‑, Notfall- und Schmerzmedizin, Schwarzwald-Baar-Klinikum, Villingen-Schwenningen & Donaueschingen, Deutschland; 9 Medizinische Fakultät, Universität Göttingen, Göttingen, Deutschland; 10 Fakultät für Gesundheit, Universität Witten/Herdecke, Witten, Deutschland

**Keywords:** Makrophagenaktivierungssyndrom-ähnliche Sepsis, Zytokinsturm, Ruxolitinib, Virale Pneumonie, Telemedizin, Macrophage activation syndrome like sepsis, Cytokine storm, Ruxolitinib, Viral pneumonia, Telemedicine

## Abstract

**Hintergrund:**

Patienten mit schwerer COVID-19-Erkrankung entwickeln eine hyperferritinämische Inflammation, ein sepsisähnliches Immundysregulationssyndrom.

**Methode:**

Retrospektive Kohortenanalyse nach Therapiestratifizierung in einer standortübergreifenden telemedizinischen Fallkonferenz. Frühzeitige, standardisierte Identifizierung von Patienten mit einem Risiko für einen schweren Verlauf (COVID-Inflammation-Score; CIS) und Intubationsvermeidung mit Schwerpunkt auf nichtinvasive Ventilation (NIV) sind Kernelemente des Behandlungsalgorithmus. Patienten mit lebensbedrohlicher Inflammation wurde ein individueller Heilversuch mit dem Immunmodulator Ruxolitinib angeboten.

**Ergebnisse:**

Zwischen 04.03.2020 und 26.06.2020 wurden 196 COVID-19-Patienten behandelt. Der Altersmedian (70 Jahre) und die Komorbidität waren im Interstudienvergleich hoch. Die Gesamtmortalität lag bei 17,3 %, wobei bei der Hälfte der verstorbenen Patienten eine A‑priori-Therapielimitierung festgelegt war. Das CIS-Monitoring der mit Ruxolitinib behandelten Hochrisikopatienten (*n* = 20) ergab nach 5, 7 und 15 Tagen eine Inflammationssuppression um 42 % (15–70), 54 % (15–77) und 60 % (15–80). In dieser Gruppe lag die Mortalität bei 20 % (4/20). Die Gesamtmortalität adjustiert auf Patienten mit intendierter Maximaltherapie lag bei 8,7 % (17/196).

**Schlussfolgerung:**

Die COVID-19-Pneumonie mit hyperferritinämischer Inflammation ist verwandt mit der Makrophagen-Aktivierungssyndrom-ähnlichen Sepsis. Eine interdisziplinäre Fallkonferenz als Qualitätsinstrument der Intensivmedizin zur Erfassung seltener sepsisähnlicher Krankheitsbilder wird vorgestellt.

## Abstract (en)

**Background:**

Patients with severe COVID-19 develop hyperferritinemic inflammation, a rare sepsis-like immune dysregulation syndrome.

**Methods:**

Stratified treatment decisions in a cross-location telemedical interdisciplinary case conference were assessed in this retrospective cohort study. A standardized treatment algorithm including continuous positive airway pressure and noninvasive ventilation was implemented. A locally developed COVID inflammation score (CIS) defined patients at risk for severe disease. Patients with life-threatening inflammation were offered off-label treatment with the immune modulator ruxolitinib.

**Results:**

Between 4 March 2020 and 26 June 2020 COVID-19 patients (*n* = 196) were treated. Median patient age (70 years) and comorbidity were high in interstudy comparison. Mortality in all patients was 17.3%. However, advance care planning statements and physician directives limited treatment intensity in 50% of the deceased patients. CIS monitoring of ruxolitinib-treated high-risk patients (*n* = 20) on days 5, 7, and15 resulted in suppression of inflammation by 42% (15–70), 54% (15–77) and 60% (15–80). Here, mortality was 20% (4/20). Adjusted for patients with a maximum care directive including ICU, total mortality was 8.7% (17/196).

**Conclusion:**

Severe COVID-19 pneumonia with hyperferritinemic inflammation is related to macrophage activation syndrome-like sepsis. An interdisciplinary intensive care teleconference as a quality tool for ICUs is proposed to detect patients with rare sepsis-like syndromes.

## Hintergrund und Fragestellung

Eine Subgruppe der mit dem SARS-CoV‑2 Infizierten zeigt keine bis milde Symptome. Etwa 14 % entwickeln schwere Krankheitszeichen mit stationärer Behandlungspflichtigkeit, weitere 5 % benötigen eine intensivmedizinische Behandlung. Die intensivmedizinische Mortalität erreicht 50 % [3]. Intensivmedizinische Aufnahmeindikation ist in der Regel die respiratorische Insuffizienz mit der Ausbildung eines akuten respiratorischen Distress-Syndroms (ARDS) auf dem Boden einer viralen Pneumonie.

Frühe Fallserien aus China wiesen bereits auf eine hyperferritinämische Inflammation im Sinne einer Zytokinsturmerkrankung als möglichen Mortalitätsfaktor hin [13, 23]. Erste Hypothesenpapiere zur Pathophysiologie des Multiorganversagens machten Anfang März 2020 auf ein aberrantes Immungeschehen aufmerksam, vergleichbar mit dem Makrophagen-Aktivierungs-Syndrom bzw. der MAS-ähnlichen Sepsis [10, 20]. Vorab wurden mittlerweile voll publizierte Behandlungsserien mit der antirheumatischen Substanz, dem Interleukin-6-Rezeptor Antikörper Tocilizumab, kommuniziert [26]. Da auch Sektionsbefunde und ein Tiermodell die Rolle der die Lunge infiltrierenden Lymphozyten und Makrophagen mit peripherer Lymphozytenaktivierung als immunogenen Pathomechanismus des Organversagens bestätigten, wurde in China der zeitlich begrenzte Einsatz von Kortikosteroiden propagiert [25]. Bereits durch das erste SARS-verursachende Coronavirus (SARS-CoV) lagen tierexperimentelle Befunde zum pulmonal getriggerten Zytokinsturm und zur medikamentösen Inflammationshemmung vor [7]. Deutsche intensivmedizinische und pneumologische Fachgesellschaften waren bis zur Publikation der Recovery-Studie zurückhaltend bezüglich einer möglichen Immunmodulation, da auf das Potenzial der verzögerten Virus-Clearance und Begünstigung sekundärer (Pilz‑)Infektionen hingewiesen wurde [12, 14].

Vor dem Hintergrund einer fehlenden Standardtherapie bei lebensbedrohlicher COVID-19-Erkrankung wurden weltweit Patienten mit individuell indizierter Immunmodulation durch selektiv Zytokin-gerichtete Substanzen (Tocilizumab, Anakinra) oder Kinaseinhibitoren des Zytokin-Signalweges JAK/STAT (Ruxolitinib, Baricitinib) zur Dämpfung der Inflammation behandelt [5, 6, 9, 17, 26].

Am Schwarzwald-Baar-Klinikum wurde am 04.03.2020 die erste SARS-CoV-2-infizierte Patientin aufgenommen und parallel ein COVID-Behandlungsalgorithmus definiert. Zentrale Säulen der Qualitätsinitiative war die strikte räumliche Trennung durch ein ausschließlich COVID-19-Patienten aufnehmenden Zweit-Standort (Lungenzentrum Donaueschingen) und die Etablierung einer standortübergreifenden, interdisziplinären COVID-19-Videokonferenz („COVID-Board“). Die intensivmedizinische Fallkonferenz wird als Modell zur Identifikation und interdisziplinären Therapieführung seltener hyperferritinämischer Verlaufsformen sepsisähnlicher Krankheitsbilder vorgestellt.

## Studiendesign und Untersuchungsmethoden

### Krankenhausweite COVID-19-SOP

Eine stadienabhängige medikamentöse Therapie mit hochdosiertem Vitamin C (1000 mg), Acetylsalicylsäure (500 mg), D‑Dimer-stratifizierter Antikoagulation, Hydroxychloroquin (600 mg bid Tag 1, 200 mg bid Tag 2–5) und Prednisolon (2 mg/kg Tag 1–3) wurde implementiert. Eine standardisierte COVID-19-Labordiagnostik wurde jeweils bei klinischer Verschlechterung zur Charakterisierung der Inflammation und Organschädigung veranlasst (Tab. 1). Diese enthielt neben den üblichen Routineparametern Serumspiegel für Interleukin‑6, den löslichen IL2-Rezeptor (sIL2R), Procalcitonin, Laktatdehydrogenase (LDH), Fibrinogen, D‑Dimer, C‑reaktives Protein (CRP) und ein Differenzialblutbild. Bezüglich der apparativen, beatmungsmedizinischen Differenzialtherapie wurden Patienten bis zu einem positiven endexpiratorischen Druck (PEEP) von 12 cmH_2_0, einem p_a_O_2_/F_i_O_2_ <90 mm Hg, inklusive Bauchlage, nichtinvasiv ventiliert (continuous positive airway pressure (CPAP) oder NIV) und auf Intermediate Care Station (IMC) behandelt, sofern dies von den Patienten toleriert wurde und nicht eine sofortige Intubation angezeigt war. Die Verlegungskriterien auf die Intensivstation (ITS) war neben einer katecholaminpflichtigen, hämodynamischen Instabilität vor allem eine fortgeschrittene intubationspflichtige respiratorische Insuffizienz.

**Table 1:** 

Kriterien	Punkte
*Radiologisch bds. Lungeninfiltrate*	**3**
CRP >20 * ULN	**2**
Ferritin >2 * ULN	**2**
Triglyzeride >1,5 * ULN	**1**
*IL‑6* *>* 3 * ULN	**1**
*Fibrinogen* *>* *ULN*	**1**
*Leukozyten* *>* *ULN*	**1**
*Lymphopenie <1,1/nL*	**2**
*Fieber >38,5* *°C*	**2**
*Gerinnungsaktivierung* DIC (D-Dimer > ULN) PTT > ULN	**1**

*ULN* Oberer Normalwert

### Therapiestratifikation nach Inflammationsgrad

**Figure 1:**
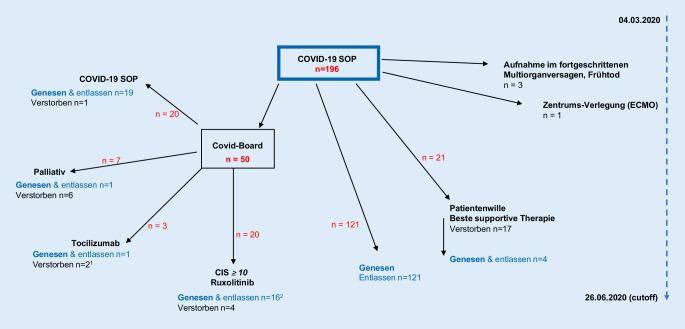

Zur Risikostratifikation wurde ein COVID-Inflammations-Score (CIS) auf dem Boden der zu diesem Zeitpunkt publizierten chinesischen COVID-Serien entwickelt (Tab. 1; [17]). Patienten mit einem CIS ≥10 (von 16 Punkten) wurden als hyperinflammatorisch eingestuft und in die interdisziplinäre, intensivmedizinische Fallkonferenz (COVID-Board) zur Indikationsprüfung möglicher immunmodulatorischer Therapieansätze eingeschlossen (Abb. 1). Voraussetzung hierfür war die Abwesenheit einer unkontrollierten sekundären Infektion, das Fehlen terminaler Organinsuffizienz (vor SARS-CoV-2-Infektion) und eine Multimorbidität mit einer COVID-19 unabhängigen Lebenszeitprognose <6 Monate.

### COVID-Board

Eine tägliche telemedizinische Konferenzschaltung zwischen Zentralklinikum und Standort Donaueschingen der Fachdisziplinen Anästhesiologie, Pneumologie, Geriatrie/Palliativmedizin, Hepatologie/Allgemeine Innere Medizin und Hämatologie/Immunologie identifizierte hyperinflammatorische Patienten und erarbeitete eine dokumentierte Therapieempfehlung.

#### Datenmanagement

Die Datenanalyse basierte auf den Patientenakten und dem Klinikinformationssystem Orbis (AGFA Health Care GmbH, Bonn, Deutschland). Die anonymisierte Datenaufbereitung erfolgte in MS Excel (Microsoft, Redmond, WA, USA).

#### Ethik

Die anonymisierte, retrospektive Analyse erfolgte auf Grundlage eines Ethikvotums der Landesärztekammer Baden-Württemberg (Nr. F‑2020-052). Die Untersuchung erfolgte gemäß der Deklaration von Helsinki.

## Ergebnisse

### Patientencharakteristika

Von 23.02.2020 bis 26.06.2020 wurden 196 Patienten mit symptomatischer COVID-19-Erkrankung am Schwarzwald-Baar-Klinikum behandelt (Tab. 2). Hiervon wurden bei klinischer Verschlechterung bzw. bei primär kritischer Erkrankung *n* = 50 Patienten im COVID-Board vorgestellt. Während das mediane Alter in der Gesamtkohorte (70 Jahre) nahezu dem in der Fallkonferenz entsprach (69 Jahre), wurden in der Fallkonferenz mehr männliche Patienten vorgestellt (*n* = 33; 66 %). Zwei Drittel der Patienten litten in beiden Kohorten unter vorbekannten Herz-Kreislauf-Erkrankungen (66 %, 70 %). Diabetes mellitus fand sich in den COVID-Board-Patienten angereichert (29 % vs. 38 %). Onkologische Erkrankungen betrafen 20 % in der Gesamtkohorte und 26 % im COVID-Board.

**Table 2:** 

Charakteristika		Gesamt *n* = 196	COVID-Board *n* = 50
Alter, Jahre, Median (Range) –		70 (0,1–95)	69 (38–85)
Männlich, no. (%)		104 (53)	33 (66)
Weiblich, no. (%)		92 (47)	17 (34)
Komorbiditäten, no. (%)			
Herz-Kreislauf-Erkrankungen		130 (66)	35 (70)
Diabetes		56 (29)	19 (38)
Lungenerkrankung		45 (23)	12 (24)
Niereninsuffizienz		41 (21)	9 (18)
Maligne Erkrankung		39 (20)	13 (26)
Chronische NIV-Behandlung, no. (%)		7 (4)	1 (2)
Behandlung, no. (%)			
Nur NIV- oder CPAP-Behandlung		46 (23)	24 (48)
Nur invasiv beatmet		8 (4)	3 (6)
NIV + invasive Beatmung		8 (4)	7 (14)
Beatmungstage, Median (Range)		9 (1–68)	11 (1–68)
ITS-Aufenhalt (%)		31 (16)	19 (38)
ITS-Aufenhalt, Tage, Median (Range) –		10 (1–63)	12 (4–63)
Hydroxychloroquin (%)		75 (38)	35 (70)
Kortikosteroide		36 (18)	27 (54)
Ruxolitinib		20 (10)	20 (40)
Tocilizumab		4 (2)	4 (8)
Temperatur Tag 1 ≥38,5 °C, no. (%) ^a^		21 (11)	8 (16)
Laborwerte Tag 1, median (Range)	Normwerte		
Ferritin, ng/ml	30–400	490 (6–3867)	928 (22–3867)
IL‑6, x ULN ^b^		–	10,88 (0,2–1417,9)
Maximaler sIL2r, U/ml ^c^	158–623	–	1519 (285–7192)
C‑reaktives Protein, mg/l	<5	53,96 (0–430)	75,04 (2,38–430)
Procalcitonin, ng/ml	<0,5	0,07 (0,01–203)	0,11 (0,01–3,54)
D‑Dimere, mg/l	0,19–0,55	1,095 (0,1–186,6)	1,32 (0,1–22,17)
Leukozyten, /nl	4–10	7,06 (1,56–31,37)	7,19 (1,97–29,93)
Lymphozyten absolut, /nl	1,2–3,4	1 (0,07–4,31)	0,9 (0,07–2,38)
Krankenhausverweildauer, Median (Range) – Tage		12 (1–71)	19 (4–71)
Mortalität (gesamt), no. (%)		34 (17,3) ^d^	13 (26)
Verstorben nach a priori Therapielimitierung		17 (50)	0
Verstorben nach therapielimitierender Entscheidung im COVID-Board		–	6 (46)
Mortalität (gesamt, ohne Pat. mit a priori Therapielimitierung), no. (%)		17 (8,7)	–
ITS-Mortalität, no. (%)^e^		14 (45,2) ^d^	9 (47,4)

*ITS* Intensivstation, *NIV* nichtinvasive Beatmung

^a^Von 186 Patienten vorhanden

^b^IL‑6 in „upper level of normal“, am Tag der ersten Vorstellung im COVID-Board

^c^Maximaler sIL2R während des Krankenhausaufenthalts

^d^Ein Patient nach Zentrumsverlegung (ECMO) auf externer ITS verstorben

^e^Ohne CPAP/NIV-beatmete IMC-Patienten

### Inflammatorische Charakterisierung der Patienten im COVID-Board

Zur Erfassung der hyperferritinämischen Inflammation wurden im COVID-Basislabor sowie bei klinischer Verschlechterung Parameter der Hyperinflammation (Differenzialblutbild, CRP, Triglyzeride, Ferritin, sIL2R, IL6, Procalcitonin) sowie der Hyperkoagulation (Quick, INR, PTT, Fibrinogen, D‑Dimer) bestimmt. Radiologische Lungenbildgebung (konventionelles Röntgen-Thorax oder Computertomographie der Lunge) sowie Fieber wurden zusätzlich für den CIS einbezogen (Tab. 2). Als Zeichen der hyperferritinämischen Inflammation und Korrelat der zentralen Rolle des Monozyten-Makrophagensystems der Lunge in der immunologischen COVID-Abwehr [21] fanden sich deutlich erhöhte Ferritinwerte bis maximal 3867 ng/ml. Ergänzend zeigte als Surrogat der lymphozytären Aktivierung der sIL2R eine im Median 2‑fache Erhöhung über den oberen Normwert (1519 U/ml (285–7192)). Beide, Ferritin und sIL2R, lagen damit deutlich unterhalb der für die hämophagozytische Lymphohistiozytose oder das Makrophagen-Aktivierungssyndrom bekannt exzessiv erhöhten Werte [4], reihen sich jedoch gut in hyperferritinämische Inflammation bei Sepsispatienten ein [18].

### Mortalität und Komorbidität

Die Gesamtkohorte zeigte eine Mortalität von 17,3 % (34/196) und eine ITS-Mortalität von 45,2 % (14/31). Siebzehn der 21 Patienten, die a priori eine beste supportive Therapie ohne intensivmedizinische Therapieeskalation wünschten, sind unter palliativmedizinischer Therapie verstorben (Abb. 1). Damit ist die um diese Patienten bereinigte Mortalität 17/196 (8,7 %). Bei 7 der 50 in das COVID-Board eingebrachten Patienten wurde eine Therapielimitierung konsentiert, hiervon sind 6 Patienten verstorben, einer wurde genesen entlassen. Drei Patienten wurden im fortgeschrittenen Multiorganversagen stationär zugewiesen und sind innerhalb von Stunden in der ZNA (*n* = 1) oder auf Intensivstation (*n* = 2) verstorben. Eine Patientin wurde zur extrakorporalen Oxygenierung (ECMO) in ein universitäres Zentrum verlegt und ist dort verstorben. Die intensivmedizinische Gesamtletalität von 45,2 % errechnet sich somit aus Patienten mit bereits bei Verlegung fortgeschrittenem respiratorischem bzw. Multiorganversagen. Bei intubationsvermeidendem Beatmungsalgorithmus wurden NIV/CPAP-Patienten der IMC nicht der ITS-Grundgesamtheit zugeordnet.

### Immunmodulatorische Therapie

Bei nahezu der Hälfte der Patienten (*n* = 20; 40 %) wurde im COVID-Board bei CIS-Werten ≥10 (Median: 12; Range: 10–14) eine Empfehlung zur Immunmodulation mit dem oralen JAK1/2-Inhibitor Ruxolitinib ausgesprochen und in der Folge nach Einholung des „informed consent“ auch umgesetzt. In die hier vorgestellte Analyse gehen die 14 Patienten der Erstpublikation zu Ruxolitinib bei SARS-CoV‑2 ein [17]. Vier der 20 (20 %) hyperinflammatorischen Hochrisikopatienten sind verstorben, 16 sind als genesen entlassen worden. Das Monitoring der Inflammation anhand klinischer Faktoren (Respiration, Fieber und des COVID-Labors) ergab nach 5, 7 und 15 Tagen eine CIS-Reduktion um 42 % (15–70), 54 % (15–77) und 60 % (15–80) als Surrogat für die Inhibition der pathologischen Immunreaktion mit sepsisähnlichem Charakter.

## Diskussion

Die COVID-19-Erkrankung führt bei einem Fünftel der SARS-CoV‑2 positiv getesteten Patienten zu einem pulmonal getriggerten Krankheitsbild. Eine Subgruppe dieser Patienten entwickelt eine hyperferritinämische Inflammation mit drohendem Multiorganversagen.

### Hyperferritinämische Inflammationssyndrome: COVID‑19 und MAS‑ähnliche Sepsis

Hyperferritinämische Inflammationssyndrome sind eine zunehmend bekannter werdende Gruppe intensivmedizinisch zu behandelnder Syndrome, zu denen u. a. die klassische Sepsis, die hämophagozytischen Syndrome HLH und MAS-HLH, die MAS-ähnliche Sepsis (MAS-like Sepsis) sowie die inflammatorischen Fiebersyndrome des konstitutionell aktivierten Inflammasoms (systemische juvenile idiopathische Arthritis, Morbus Still, Cryopyrin-assoziierte periodische Syndrome) und das iatrogene Zytokin-Release-Syndrom (CRS) gerechnet werden [8, 16]. Verschleppte Diagnosen seltener Inflammationssyndrome beruhen auf den häufig überlappenden Leitbefunden [20].

Für die hämophagozytische Lymphohistiozytose (HLH) konnte durch Analyse der Ferritinwerte aus einem großen Sepsiskollektiv retrospektiv eine 78 % Fehldiagnoserate (7/9 Patienten) bei eigentlich vorliegender HLH eruiert werden [19]. Für die Subgruppe der MAS-ähnlichen Sepsis konnte post hoc aus einem großen, prospektiv mit dem Interleukin-1-Rezeptor-Antagonisten Anakinra behandelten Sepsiskollektiv ein 50 % verbessertes Gesamtüberleben ausschließlich für MAS-like Patienten bei ansonsten negativem Studienergebnis für Anakinra berichtet werden [24]. Kürzlich wurden die positiven Ergebnisse der prospektiven britischen RECOVERY-Studie zu 10-tägiger niedrig dosierter Dexamethason-Therapie bei schwerer COVID-19-Erkrankung berichtet, welche eine Überlegenheit bzgl. Gesamtüberleben in der Dexamethason-Gruppe für O_2_- und beatmungspflichtige Patienten zeigte [12].

Der von uns beschriebene Ansatz, über gezielte Inhibition des JAK/STAT-Signalweges mit Ruxolitinib den Zytokinsturm der COVID-19-Erkrankung molekular zu bremsen, zeigt einen weiteren vielversprechenden Ansatz der personalisierten Inflammationshemmung der COVID-19-Hyperferritinämie [5, 17]. Dabei ist die prospektive Risikostratifizierung mittels des neu entwickelten Score-Systems (CIS) hervorzuheben (Tab. 1). Der CIS wird nun in einer prospektiven multizentrischen COVID-19-Therapiestudie (RuxCoFlam, NCT04338958) deutschlandweit validiert. Die in der vorgelegten Fallserie eruierte Gesamtmortalität der COVID-19-Patienten von 17 % bzw. 8,7 % unter Ausschluss der a priori definierten Therapiezielbegrenzung, ist im Vergleich mit den international publizierten Zahlen vor dem Hintergrund hoher Interstudien-Variabilität bzgl. Eingangskriterien (Ausschluss Frühtod? Ausschluss von Patienten mit a priori Therapielimitation? Versorgungsstruktur, Altersspektrum und Komorbiditäten) sehr vorsichtig einzuordnen [11, 22, 27].

### Fachärztliche Interdisziplinarität auf Intensivstation

**Figure 2:**
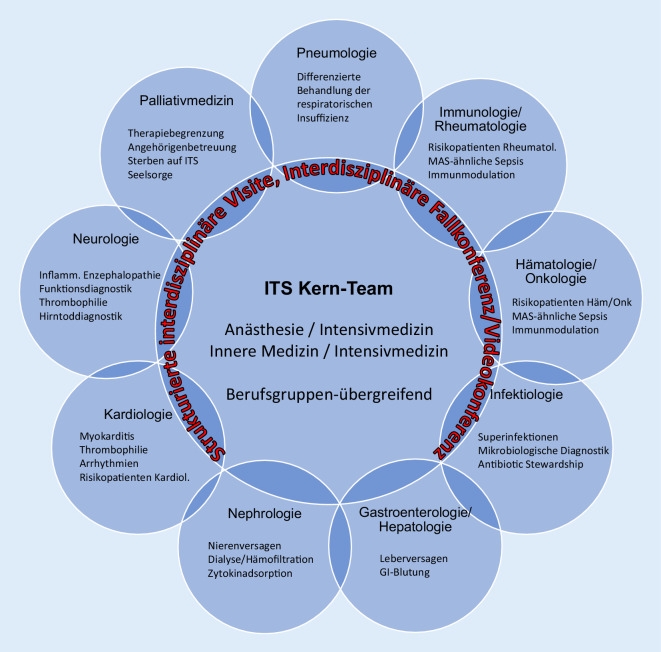

Die weltweit gesammelten Erfahrungen mit dem intensivmedizinischen Management von COVID-19 mit hyperferritinämischer Inflammation weisen auf das potenzielle Risiko einer verpassten Immunmodulation bei sepsisähnlichen Krankheitsbildern. Hier berichten wir von einer interdisziplinären Fallbesprechung (COVID-Board) unter kollegialer Leitung der Beatmungsmedizin (Pneumologie) und Intensivmedizin (Anästhesie) mit dem Ziel, gemeinsam die diagnostische Vigilanz zu erhöhen und die Differenzialtherapie der sepsisähnlichen Hyperinflammation durch gezielte Immunmodulation zu optimieren (Abb. 2). Hierfür zeigt die Literatur unseres Wissens noch keine Vorbildstrukturen. Im Wesentlichen konzentriert sich die Interdisziplinarität auf die Intensivtherapie und die kollegiale Zusammenarbeit zwischen den Berufsgruppen des Kernteams [1].

Unsere interdisziplinären Erfahrungen in der Behandlung der COVID-19-Erkrankung unterstützen die Notwendigkeit einer fachärztlichen Interdisziplinarität bei ausgesuchten Patienten mit komplexen Krankheitsbildern. Gelebte Interdisziplinarität beruht in vielen Bereichen derzeit noch auf engagierten Einzelpersönlichkeiten. Sie erscheint den Autoren jedoch unerlässlich für gegenseitigen Wissensgewinn, die ganzheitliche Betrachtung eines komplexen Krankheitsbildes und auch die Entwicklung entsprechender Studienkooperationen.

Einen beispielgebenden Lernprozess zur Interdisziplinarität stieß die Deutsche Krebsgesellschaft (DKG) durch das Zertifikat „Onkologisches Zentrum“ an. Interdisziplinarität wurde messbar und wirkte qualitätsverbessernd [15]. Wir werben daher für eine verbesserte Zusammenarbeit des intensivmedizinischen Kernteams mit Fachexpertise aus der Immunologie und Infektiologie. COVID-19 mit der aberranten, pulmonal getriggerten Immunantwort und der hyperferritinämischen Inflammation könnte hier als modellhafter Katalysator wirken. Die interdisziplinäre Fallkonferenz wäre mit eingebrachten Patienten als Kennzahl für das Peer Review ein geeignetes Instrument.

### Telemedizin in der Intensivmedizin

COVID-19 wirkt auch als Katalysator für eine Kultivierung der klinischen Videokonferenzen, ein weiteres mögliches Qualitätsinstrument einer interdisziplinären Intensivmedizin, wie die vorgelegte Studie unseres Zwei-Standort-Klinikums zeigt. Telemedizin, als standortübergreifende Betreuung von Intensivpatienten, wird in der Literatur eher als Ausbildungsinstrument beschrieben, als eine Plattform für die fachärztlich interdisziplinäre Fallkonferenz [2]. Insbesondere für Häuser ohne ansässige Spezialdisziplinen (Onkologen/Pneumologen/Infektiologen) wäre das telemedizinische Modell ein zu evaluierendes Qualitätsinstrument.

## Schlussfolgerung

Mit der gezielten Inhibition des COVID-19-Zytokinsturms durch den JAK1/2-Inhibitor Ruxolitinib konnte in einem selektierten Patientengut eine effektive Inflammationskontrolle erreicht werden. Den komplexen, intensivmedizinischen Aspekten des COVID-19-Managements wurde in der vorgelegten monozentrischen Fallserie eines kommunalen Klinikums der Zentralversorgung durch telemedizinische, interdisziplinäre Fallbesprechung Rechnung getragen. Die Autoren schlagen vor, bei Patienten mit hyperferritinämischer Inflammation und Sepsisverdacht die immunologische und infektiologische Fachexpertise strukturell noch stärker in die klassische intensivmedizinische Therapie zu integrieren. Prospektive Untersuchungen sind zur Validierung der postulierten Qualitätsverbesserung erforderlich.

## Fazit für die Praxis

- Die COVID-19-Pneumonie verursacht eine hyperferritinämische Hyperinflammation.
- Die hyperferritinämische Inflammation wurde mit einem neu entwickelten COVID-19-Inflammations-Score (CIS) charakterisiert.
- Die mit der MAS-ähnlichen Sepsis verwandte Hyperinflammation wurde bei fehlendem Therapiestandard als individueller Heilversuch mit dem JAK1/2-Tyrosinkinase-Inhibitor Ruxolitinib behandelt.
- Die interdisziplinäre Therapiefindung und das Monitoring der Patienten wurde standortübergreifend in einer telemedizinischen Fallkonferenz strukturiert.
- Die intensivmedizinische Entscheidungsfindung zur Erkennung und Behandlung seltener Krankheitsbilder bedarf den Instrumenten der strukturierten Interdisziplinarität.
